# Gut microbial metabolites in inflammatory bowel disease: immunological mechanisms regulating Treg/Th17 balance and therapeutic potential

**DOI:** 10.3389/fimmu.2026.1780865

**Published:** 2026-02-25

**Authors:** He Chen, Shaochen Yu, Mengjie Zhang, Beibei Tian, Langlang Yang, Jian Lu

**Affiliations:** 1 Department of Internal Medicine, Beijing Jishuitan Hospital Guizhou Hospital, Guiyang, Guizhou, China; 2 Department of Emergency and Critical Care Medicine, Chuzhou Integrated Traditional Chinese and Western Medicine Hospital, Chuzhou, Anhui, China; 3 Department of Gastroenterology, The First Affiliated Hospital of Anhui Medical University, Hefei, Anhui, China

**Keywords:** gut microbiota, immunomodulation, inflammatory bowel disease, microbial metabolites, regulatory T cells, T helper 17 cells

## Abstract

Inflammatory bowel disease (IBD) is a chronic inflammatory disorder resulting from a combination of genetic susceptibility, environmental factors, and an abnormal immune response of the intestinal immune system to commensal microorganisms. The gut microbiota and its metabolites play a pivotal role in maintaining intestinal immune homeostasis. Recent advances indicate that dysbiosis of the microbiota is accompanied by alterations in its metabolic functions. Abnormal levels of key metabolites, particularly short-chain fatty acids (SCFAs), tryptophan derivatives, and secondary bile acids, are closely associated with the pathogenesis of IBD. These metabolites act as G protein-coupled receptor ligands, nuclear receptor ligands, or epigenetic modifiers, deeply involved in the differentiation, function, and dynamic balance between regulatory T cells (Tregs) and T helper 17 cells (Th17). Disruption of the Treg/Th17 balance is a central driver of intestinal immune inflammation in IBD. This review systematically explores the molecular networks through which major microbial metabolites regulate the differentiation and function of Treg and Th17 cells, including their profound effects on cellular metabolic reprogramming, the epigenetic landscape, and the local immune microenvironment. Furthermore, it analyzes how the disturbance of the microbial metabolome in the pathological state of IBD leads to the attenuation of beneficial immunoregulatory signals and the generation of potential pro-inflammatory signals, thereby contributing to a vicious cycle of immune tolerance deficiency and chronic inflammation. Based on these mechanisms, this article evaluates therapeutic strategies targeting the microbiota-metabolism-immune axis, such as dietary interventions, probiotics/prebiotics, postbiotics, engineered bacterial therapies, fecal microbiota transplantation, and small-molecule receptor modulators, discussing their current status and challenges. Finally, the limitations of current research are outlined, and future directions are proposed, including the use of integrated multi-omics analyses, spatial biology technologies, and organoid models to advance the development of personalized precision medicine.

## 1 Introduction

Inflammatory bowel disease (IBD), primarily comprising Crohn’s disease (CD) and ulcerative colitis (UC), is characterized by chronic, relapsing intestinal inflammation. Its global incidence is continuously rising, posing a significant public health burden (39815450). The etiology of IBD is not fully elucidated but is widely considered an inappropriate immune response to intestinal commensal microorganisms in genetically susceptible individuals triggered by specific environmental factors (1). Genome-wide association studies have identified numerous IBD-associated risk loci involving innate immunity (e.g., NOD2), autophagy (e.g., ATG16L1), the Th17 cell pathway (e.g., IL23R, STAT3), and epithelial barrier function, among others (2). However, genetic factors account for only a portion of disease susceptibility, highlighting the central role of environmental factors in disease development (3). Among various environmental factors, the gut microbiota, as a critical interface connecting the external environment with the host immune system, has garnered increasing attention.

Early research focused on characteristic alterations in the gut microbiota composition of IBD patients, termed dysbiosis, manifested as reduced microbial diversity, a decrease in bacteria with potential beneficial functions (e.g., butyrate-producing *Faecalibacterium prausnitzii*), and a relative increase in bacteria with potential pro-inflammatory tendencies (4, 5). However, attributing disease simply to an increase or decrease of specific bacteria is challenging due to the complex causality between microbiota composition and disease phenotype, often confounded by the reverse influence of the inflammatory state itself. Consequently, the research focus has gradually shifted from describing changes in microbial species composition to understanding the functional output of the microbiota—namely, the metabolites they produce (6). The gut microbiota can be viewed as a vast “biochemical reactor”, metabolizing host-indigestible dietary components (e.g., dietary fiber, protein) and endogenous substances (e.g., bile acids) into a diverse array of small-molecule compounds with varied bioactivities. These metabolites constitute a “chemical language” continuously sensed by host cells, capable of crossing or influencing the intestinal mucosal barrier to interact directly or indirectly with host immune cells, thereby profoundly shaping the development, differentiation, and function of the immune system. This shift in understanding marks a transition from an ecological description to a functional analysis of host-microbe symbiosis, providing more precise targets for mechanistic disease intervention.

Within the complex intestinal immune network, the balance among CD4+ T lymphocyte subsets, particularly between immunosuppressive regulatory T cells (Tregs) and pro-inflammatory T helper 17 (Th17) cells, is crucial for maintaining mucosal immune tolerance and effective defense. Treg cells, expressing the transcription factor Foxp3, secrete inhibitory cytokines like IL-10 and TGF-β, and employ cell-contact-dependent mechanisms (e.g., expressing CTLA-4 to compete for CD80/CD86), actively suppressing the overactivation of effector T cells, thus forming the cornerstone of intestinal immune tolerance (7). Th17 cells produce cytokines such as IL-17A, IL-17F, and IL-22, playing vital roles in defending against extracellular pathogens and maintaining epithelial integrity (8). However, aberrant activation and expansion of Th17 cells are closely linked to various autoimmune and inflammatory diseases, including IBD. During active IBD, the intestinal mucosa often exhibits hyperactivity of Th17 cells and related pro-inflammatory cytokine networks, while Treg cell numbers or function are often relatively or absolutely deficient. This imbalance is considered a core pathological driver of chronic intestinal inflammation (9). Therefore, investigating how gut microbial metabolites precisely regulate the Treg/Th17 balance has become a key scientific question for understanding IBD immunopathogenesis and identifying novel therapeutic targets.

This review aims to systematically summarize and critically integrate advances in this field. First, it outlines the immunomodulatory properties of key microbial metabolites and their target receptors. Second, it delves into the molecular networks through which these metabolites integratively regulate the fate decisions of Treg and Th17 cells by influencing cellular metabolism, epigenetic modifications, and antigen-presenting cell function. Subsequently, it analyzes in detail how dysbiosis of microbial metabolic functions in IBD leads to the collapse of these regulatory networks. Then, based on mechanistic understanding, it evaluates the potential and challenges of various therapeutic strategies targeting this axis. Although the immunomodulatory roles of short-chain fatty acids (SCFAs), aryl hydrocarbon receptor (AhR) ligands, and bile acids have been extensively reported, core challenges remain: how these three classes of metabolites form hierarchical, temporal, or redundant regulatory networks *in vivo* to collectively maintain homeostasis; their interaction patterns during IBD-associated dysregulation; and how to translate complex metabolomic information into actionable personalized therapeutic targets.

While numerous reviews have addressed the role of gut microbiota composition in Th17/Treg imbalance in IBD, a metabolite-centered perspective offers distinct conceptual and translational advantages. First, microbial metabolites represent the functional output of the microbiota, serving as direct chemical messengers that mediate host–microbe crosstalk. Unlike taxonomic shifts, which may not consistently predict functional changes, metabolites provide a more precise and mechanistic link between microbial ecology and host immunology. Second, metabolites often act through well-defined receptors (e.g., GPCRs, AhR, nuclear receptors) and modulate specific intracellular pathways (e.g., HDAC inhibition, mTOR signaling, epigenetic remodeling), offering clearer targets for therapeutic intervention. Third, focusing on metabolites allows the integration of dietary, microbial, and host metabolic factors into a unified “metabolism–immune” axis, facilitating the development of metabolite-based diagnostics, dietary strategies, and small-molecule therapies. By elucidating how metabolite networks—rather than individual microbial taxa—orchestrate immune balance, this review aims to advance the field from descriptive ecology to actionable functional mechanism, thereby informing more precise and effective therapeutic approaches for IBD.

## 2 Immunomodulatory properties of core microbial metabolites

The diversity of gut microbial metabolites underpins their functional diversity. Based on chemical properties and precursor sources, these metabolites can be categorized into several classes. Among them, SCFAs, tryptophan derivatives, and secondary bile acids are three extensively studied and well-evidenced immunomodulatory molecules. They not only reflect the metabolic state of the microbiota but also serve as key mediators in the “chemical dialogue” between the microbiota and the host immune system, exerting broad and precise immunoregulatory functions by acting on specific host cell receptors or directly participating in intracellular biochemical processes.

### 2.1 SCFAs: multifunctional metabolic and signaling molecules

SCFAs, primarily acetate, propionate, and butyrate, are the main end products of dietary fiber fermentation by colonic anaerobic bacteria (e.g., *Clostridium* clusters IV and XIVa) (10). In the colonic lumen, SCFA concentrations can reach millimolar levels. Butyrate is the preferred energy source for colonocytes, accounting for 60-70% of their energy expenditure, and is crucial for maintaining epithelial cell viability, tight junction protein expression, and mucus secretion, forming an essential foundation for intestinal barrier function (11). Beyond this metabolic/nutritive role, SCFAs are critical immunomodulatory signaling molecules, with mechanisms of action exhibiting multidimensional features.

First, SCFAs act as potent inhibitors of histone deacetylases (HDACs). Butyrate and propionate specifically inhibit the activity of Class I and IIa HDACs, leading to increased intracellular histone acetylation, open chromatin structure, and subsequent modulation of gene transcription programs (12, 13). This epigenetic regulatory mechanism is particularly crucial for inducing and stabilizing the expression of Foxp3, the key transcription factor for Treg cells, as the Foxp3 locus contains multiple conserved non-coding sequence (CNS) enhancer regions whose chromatin accessibility is strictly regulated by histone acetylation status.

Second, SCFAs serve as natural ligands for G protein-coupled receptors (GPCRs). SCFAs are endogenous agonists for receptors such as GPR41 (FFAR3), GPR43 (FFAR2), and GPR109A (HCAR2) (14). These receptors are widely expressed in the intestine on intestinal epithelial cells, lamina propria immune cells (e.g., macrophages, dendritic cells, neutrophils), and T cells. For example, butyrate activation of GPR109A on macrophages and dendritic cells can induce these cells to produce high levels of the anti-inflammatory cytokine IL-10 while promoting the synthesis of immunomodulatory retinoic acid (RA) (15). This metabolite-triggered receptor signaling can indirectly yet effectively promote local Treg differentiation and suppress excessive inflammatory responses. Activation of GPR43 has also been shown to inhibit neutrophil and monocyte chemotaxis and activation, alleviating inflammation (16). It is noteworthy that the immunological outcomes of SCFA-GPCR signaling are predominantly anti-inflammatory within the intestinal microenvironment, albeit with cell-type specificity and contextual nuances. The primary SCFA receptors discussed here—GPR43 (FFAR2), GPR41 (FFAR3), and GPR109A (HCAR2)—are generally categorized as mediating anti-inflammatory or immunoregulatory signals in the gut. Their activation on intestinal epithelial cells, macrophages, and dendritic cells typically promotes an anti-inflammatory state by enhancing the production of IL-10, TGF-β, and retinoic acid, while inhibiting the recruitment and activation of neutrophils. However, the functional consequence of signaling through a given GPCR subtype can indeed vary depending on the cellular context. For instance, while GPR109A activation on macrophages/dendritic cells drives an anti-inflammatory program, its role in other cell types (e.g., intestinal epithelial cells) may involve additional barrier-strengthening functions. Furthermore, the net effect may be influenced by the inflammatory milieu; in established, severe inflammation, receptor expression or downstream signaling pathways might be altered, potentially dampening these beneficial responses. Thus, while the SCFA-GPCR axis is a cornerstone of intestinal immune homeostasis, its precise effects are integrated from signals across multiple cell types within the tissue microenvironment. The key SCFAs, their primary GPCR targets, expressing cell types, and major immunological effects are summarized in **Table 1**.

**Table 1:** Immunomodulatory roles of major short-chain fatty acids (SCFAs) through G protein-coupled receptor (GPCR) signaling in the intestine.

SCFA	Primary GPCR Target(s)	Major Intestinal Cell Types Expressing the Receptor	Predominant Immunological Effect(s) in the Intestinal Context
Acetate	GPR43 (FFAR2), GPR41 (FFAR3)	Intestinal epithelial cells (IECs), Lamina propria immune cells (e.g., neutrophils, macrophages, dendritic cells), Enteroendocrine cells	Anti-inflammatory. Inhibits neutrophil chemotaxis and activation via GPR43; promotes IL-10 and PGE2 production; enhances epithelial barrier function.
Propionate	GPR43 (FFAR2), GPR41 (FFAR3)	IECs, Lamina propria immune cells (macrophages, dendritic cells, T cells), Enteroendocrine cells	Anti-inflammatory / Immunoregulatory. Similar to acetate; strongly induces IL-10 and RA production in dendritic cells/macrophages; promotes Treg differentiation indirectly via shaping APC function.
Butyrate	GPR109A (HCAR2), GPR41 (FFAR3)	Colonic epithelial cells, Lamina propria immune cells (macrophages, dendritic cells)	Strongly Anti-inflammatory / Tolerogenic. Activation of GPR109A on macrophages/dendritic cells induces IL-10 and RA; crucial for maintaining anti-inflammatory tone and promoting Treg responses. Also serves as a primary energy source for colonocytes, reinforcing barrier integrity.

The effects summarized here represent the predominant roles under homeostatic or resolving inflammatory conditions. Signaling outcomes can be modulated by receptor expression levels, concentration gradients, and the presence of other inflammatory signals in the microenvironment.

Third, SCFAs directly modulate the metabolic state of immune cells. Immune cells can actively take up SCFAs and integrate them into their own metabolic networks. Butyrate has been shown to inhibit the activity of the mammalian target of rapamycin complex (mTORC) (17). mTOR signaling activation is required for Th17 cell differentiation and their associated glycolytic metabolic program (18). Therefore, by inhibiting mTOR, butyrate can suppress Th17 development at the metabolic source. Beyond these roles, SCFAs may influence the cellular pools of metabolic intermediates like acetyl-CoA (19), further linking cellular metabolic state to gene expression regulation. Acetyl-CoA is not only a key intermediate in energy metabolism but also the direct substrate for histone acetylation. Thus, SCFAs may play a dual role in epigenetic regulation as both substrate suppliers and enzyme activity regulators.

### 2.2 Tryptophan metabolites: the complex regulatory network of the AhR pathway

Tryptophan, an essential amino acid, is metabolized primarily via the host cell-dominated kynurenine pathway and the microbiota-dominated indole pathway. Bacteria like *Lactobacillus* and *Bifidobacterium* can convert tryptophan into various derivatives such as indole, indole-3-acetic acid, indole-3-aldehyde, and indole-3-propionic acid via tryptophanase. These indole compounds are high-affinity natural ligands for the AhR (20). AhR is a ligand-activated transcription factor belonging to the basic helix-loop-helix (bHLH)/PAS protein family, expressed in various immune cells, including Th17 cells, Treg cells, type 3 innate lymphoid cells (ILC3s), and dendritic cells. The immunomodulatory effects of AhR signaling exhibit significant context dependency, making it a complex immune calibrator.

In Th17 cells, AhR activation participates in their differentiation process and potently induces the production of the protective cytokine IL-22 (21). IL-22, a member of the IL-10 cytokine family, primarily targets epithelial cells, promoting their proliferation and regeneration, stimulating antimicrobial peptide expression (e.g., defensins, RegIII family proteins), and enhancing mucus secretion, thereby being crucial for maintaining epithelial barrier function. Notably, AhR signaling is believed to help confer certain regulatory properties to Th17 cells (22). Under specific cytokine milieus (e.g., TGF-β and IL-6), the presence of AhR ligands can prompt Th17 cells to co-express IL-17A and IL-10, forming an inhibitory Th17 cell subset, or inhibit their conversion into a highly pathogenic phenotype producing GM-CSF and IFN-γ (23–25). Such AhR-dependent Th17 cells exhibit lower pro-inflammatory potential and are more inclined to play protective roles at tissue barriers.

Moreover, AhR activation can also enhance Treg cell function or stability under certain conditions (26). Some studies indicate that AhR ligands can promote IL-10 production by Treg cells or stabilize Foxp3 expression through interactions with other signaling pathways (27). AhR activation in dendritic cells can induce TGF-β production and upregulate indoleamine 2, 3-dioxygenase (IDO) expression (28). IDO inhibits T cell proliferation and promotes Treg generation by depleting tryptophan and generating kynurenine metabolites, thereby regulating immune tolerance at an upstream level (29). In IBD patients, AhR agonist activity in intestinal contents is often reduced, likely due to a decrease in indole-producing bacteria or altered tryptophan metabolism, thereby weakening the normal regulatory role of the AhR pathway on immune cells and disrupting the Th17/Treg balance and epithelial barrier integrity (30).

### 2.3 Secondary bile acids: bridging digestive metabolism and immunomodulation

Bile acids are synthesized from cholesterol in the liver, conjugated with glycine or taurine to form primary bile acids (mainly cholic acid and chenodeoxycholic acid), secreted into the intestine, and play key roles in fat digestion and absorption. Approximately 95% of bile acids are actively reabsorbed in the distal ileum via the apical sodium-dependent bile acid transporter (ASBT), entering the enterohepatic circulation. The remaining ~5% enter the colon, becoming substrates for microbial metabolism. Colonic bacteria (e.g., *Bacteroides, Clostridium, Lactobacillus*) convert primary bile acids into secondary bile acids like deoxycholic acid (DCA), lithocholic acid (LCA), and their various derivatives through enzymes such as bile salt hydrolase and 7α-dehydroxylase (31).

For a long time, secondary bile acids were mainly considered byproducts of digestion. However, research over the past two decades has revealed them as important signaling molecules, primarily regulating host metabolism, inflammation, and immune function by activating the nuclear receptor FXR and the membrane receptor TGR5 (GPBAR1) (32). FXR is highly expressed in the liver and intestine, regulating bile acid synthesis, lipid, and glucose metabolism. TGR5 is widely expressed in various cells, including enteroendocrine cells, immune cells, and neurons; its activation stimulates glucagon-like peptide-1 (GLP-1) secretion, increases energy expenditure, and exerts anti-inflammatory effects.

Recent groundbreaking studies have found that specific secondary bile acid isomers can bypass these classical receptors to directly regulate T cell differentiation and function. For instance, 3-oxolithocholic acid (3-oxoLCA) was shown to directly enter T cells, bind to the ligand-binding domain of RORγt—the key transcription factor for Th17 cells—inducing conformational changes that inhibit RORγt’s transcriptional activity, ultimately reducing IL-17A production (33). Conversely, another isomer, isoallolithocholic acid (isoalloLCA), was found to specifically promote Treg cell differentiation by inducing mitochondrial oxidative stress and activating Foxp3 expression (34). These findings indicate that the composition of the bile acid pool, shaped by host genetics, diet, and microbial metabolic capacity, is far from homogeneous. It contains specific chemical species with precise immunomodulatory instructions, constituting a dynamic and complex chemical signal library regulating local intestinal immune balance. Bile acid metabolism abnormalities common in IBD patients, such as an increased proportion of primary bile acids and altered secondary bile acid profiles, may directly contribute to immune imbalance by changing the proportions of these active molecules (35). Furthermore, bile acid signaling via TGR5 can inhibit NLRP3 inflammasome activation in macrophages, reducing the release of pro-inflammatory cytokines like IL-1β, thereby modulating inflammation at the innate immune level (36).

## 3 Integrated molecular mechanisms of metabolite-mediated regulation of Treg/Th17 balance

**Figure 1:**
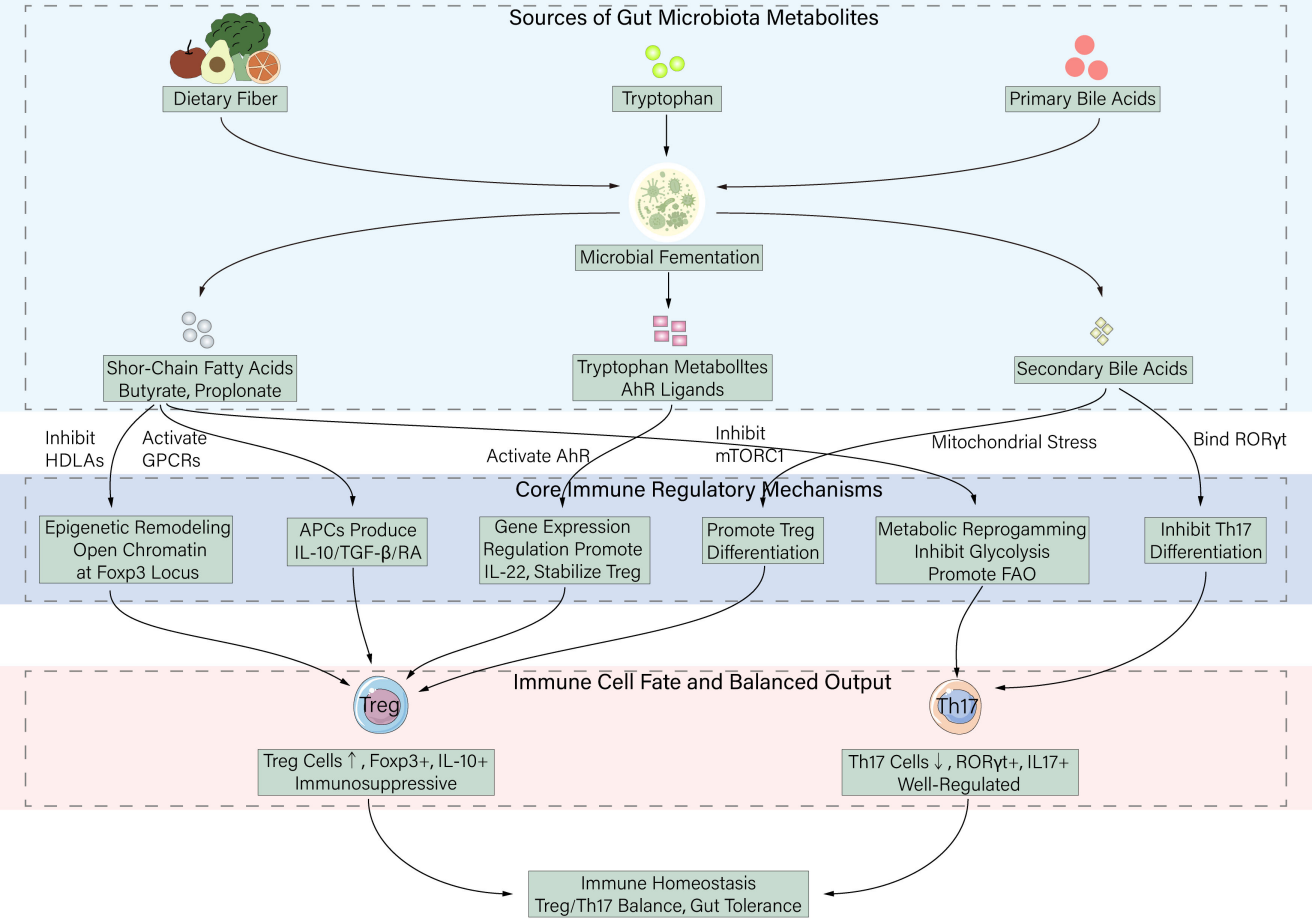
Integrated mechanisms by which gut microbiota-derived metabolites regulate Treg/Th17 balance under homeostatic conditions. Under homeostatic conditions, gut microbiota ferment dietary fiber, tryptophan, and primary bile acids to produce three key classes of metabolites: short-chain fatty acids (SCFAs, e.g., butyrate and propionate), tryptophan metabolites (AhR ligands), and secondary bile acids (e.g., 3-oxoLCA and isoalloLCA). These metabolites regulate immune cell differentiation and function through integrated mechanisms: (1) SCFAs act as HDAC inhibitors to promote Foxp3 expression, activate GPCRs to induce anti-inflammatory cytokines (IL-10, TGF-β, RA) from APCs, and inhibit mTORC1 to suppress glycolysis while promoting FAO; (2) AhR ligands activate AhR signaling to promote IL-22 production and stabilize Tregs; (3) Secondary bile acids directly inhibit RORγt (suppressing Th17) or induce mitochondrial stress (promoting Tregs). Collectively, these mechanisms promote Treg differentiation/function while restraining pathogenic Th17 responses, maintaining intestinal immune tolerance and homeostasis.

The regulation of the Treg/Th17 balance by microbial metabolites is not achieved through isolated, linear signaling pathways but rather constitutes a multi-layered, intertwined integrative network. The core logic of this network lies in metabolites serving as chemical carriers of environmental signals, translating extracellular information into persistent changes in intracellular gene expression programs. This transformation is primarily realized through two complementary levels: reprogramming the intrinsic state (metabolism and epigenetics) of T cells themselves, and shaping antigen-presenting cell (APC) function to alter the local immune microenvironment (**Figure 1**).

### 3.1 Coupling of metabolic reprogramming and epigenetic modification

T cell differentiation, proliferation, and functional execution are tightly coupled to their metabolic state. Different T cell subsets prefer distinct metabolic programs to support their specific functional demands. Th17 cell differentiation and effector function heavily rely on aerobic glycolysis (preferring glycolysis even under oxygen-sufficient conditions) and glutaminolysis (37). This metabolic switch is primarily driven by the phosphatidylinositol 3-kinase (PI3K)-AKT-mTORC1 signaling axis (38). mTORC1 activation not only promotes the expression of glycolysis-related genes (e.g., glucose transporter GLUT1, hexokinase 2) but also stabilizes hypoxia-inducible factor-1α (HIF-1α) (39), which further promotes glycolysis and directly participates in the transcriptional regulation of genes like IL-17 (40). Another requirement for Th17 differentiation is fatty acid synthesis to support the membrane synthesis needed for their rapid proliferation.

In stark contrast, Treg cells preferentially utilize oxidative phosphorylation and fatty acid oxidation (FAO) to generate energy (41). This metabolic mode produces more reactive oxygen species (ROS), but Treg cells highly express antioxidant enzymes to adapt. More importantly, acetyl-CoA generated from FAO is a crucial substrate for histone acetylation, aligning with the need for Treg cells to maintain high expression of specific genes like Foxp3 and IL-10 (42). AMP-activated protein kinase (AMPK) is a key regulator promoting FAO and mitochondrial biogenesis and typically antagonizes mTORC1 signaling (43).

Microbial metabolites can directly and profoundly intervene in these metabolic switches. Taking butyrate as an example, it inhibits Th17-favored metabolic programs through a dual mechanism: on one hand, as an HDAC inhibitor, it may indirectly affect the expression of metabolism-related genes; on the other hand, it directly inhibits mTORC1 activity, thereby shutting down the key signal driving glycolysis and Th17 differentiation (44). Concurrently, butyrate may promote mitochondrial function and FAO by activating AMPK or serving as a substrate for β-oxidation (45, 46), thereby supporting the Treg metabolic phenotype. This shift in metabolic state is not merely a change in energy supply; it directly influences the abundance of key metabolic intermediates used for biosynthesis and signaling, such as acetyl-CoA, NAD+, α-ketoglutarate (α-KG), and S-adenosylmethionine (SAM). These molecules themselves are important cofactors or substrates for epigenetic modifying enzymes like histone acetyltransferases (HATs), demethylases (e.g., KDM, TET), and methyltransferases. Therefore, by altering the cellular metabolite pool, microbial metabolites tightly link cellular metabolic state to the shaping of the epigenetic landscape, achieving coherent regulation from metabolism to gene expression. For instance, high levels of α-KG promote TET enzyme activity, leading to DNA demethylation and gene activation (47), which may facilitate Foxp3 locus demethylation and stable expression.

It is noteworthy that metabolic reprogramming and epigenetic modification are not a unidirectional “cause-and-effect” relationship but constitute a dynamic feedback loop. For example, SCFA-induced Foxp3 expression not only depends on their HDAC inhibitory activity (48), but Foxp3 itself can, in turn, regulate Treg metabolic programs, such as upregulating FAO-related genes (49). This bidirectional interlocking mechanism ensures Treg lineage functional stability. Similarly, while stabilizing Treg function, AhR ligands may also consolidate their epigenetic state by modulating cellular metabolism. Future research requires single-cell multi-omics technologies to spatiotemporally resolve the establishment process of this metabolism-epigenetics feedback loop and its specific disruption points in IBD.

### 3.2 Directed remodeling of the epigenetic landscape

Epigenetic modifications serve as a key bridge connecting dynamic environmental signals to relatively stable cell fate decisions. As the most direct environmental chemical input, microbial metabolites are important epigenetic modifiers capable of directionally remodeling the T cell epigenome, thereby locking in their functional lineage.

The HDAC inhibitory effect of SCFAs is a core epigenetic mechanism for inducing Treg differentiation (50). Butyrate and propionate, by inhibiting Class I and IIa HDACs, lead to hyperacetylation of histones H3 and H4 at specific gene loci (e.g., H3K9ac, H3K27ac). These acetylation marks are typically associated with active transcriptional enhancers and promoters. For Treg cells, the Foxp3 locus contains multiple CNS regions, with CNS1, CNS2, and CNS3 being key regulatory areas. CNS1 is enriched with TGF-β and retinoic acid response elements, particularly important for peripherally induced Treg (pTreg) differentiation; CNS2 is associated with the long-term stable maintenance of Foxp3 expression. SCFA-induced histone hyperacetylation significantly enhances the chromatin accessibility of these CNS regions, allowing transcription activators (e.g., SMAD3, NFAT, retinoic acid receptor RAR/RXR) to bind effectively, thereby robustly driving Foxp3 transcription initiation and sustained expression (51). Beyond Foxp3, SCFAs can also promote histone acetylation of other Treg functional genes like IL-10, enhancing their suppressive function (52).

Beyond histone modifications, DNA methylation is another crucial epigenetic layer regulating T cell fate. DNA methylation is typically associated with gene silencing, while DNA demethylation (via TET protein-catalyzed 5-methylcytosine oxidation) is linked to gene activation. α-KG is an essential cofactor for TET dioxygenases, and its intracellular level influences TET enzyme activity (53). Certain microbial metabolites or the host metabolic states they influence may indirectly affect the DNA methylation status of the Foxp3 locus by regulating the ratio of metabolites like α-KG/succinate, further stabilizing its expression. For example, succinate can competitively inhibit α-KG-dependent dioxygenases (including TET and histone demethylases), potentially solidifying an inhibitory epigenetic state unfavorable for Treg differentiation (54). Therefore, microbial metabolism, through metabolic intermediates, directly influences the activity of epigenetic modifying enzymes, constituting a metabolism-epigenetics axis.

### 3.3 Shaping antigen-presenting cell function and setting the immune microenvironment

*In vivo*, the differentiation direction of naïve CD4+ T cells is not solely determined by intrinsic signals but, more critically, by the local microenvironment in which they are activated—specifically, the co-stimulatory signals and cytokine combinations provided by APCs. The intestinal lamina propria is rich in dendritic cells (DCs) and macrophages, which are the first immune cells to encounter microbial metabolites from the gut lumen. Microbial metabolites can educate these APCs, thereby setting the tone for the local immune response.

SCFAs (via GPR109A, GPR43) and AhR ligands can induce intestinal APCs to adopt a “tolerogenic” or “anti-inflammatory” phenotype. Molecular features include: upregulating the secretion of anti-inflammatory cytokines IL-10 and TGF-β; enhancing retinoic acid (RA) synthesis capacity by upregulating the key enzyme retinaldehyde dehydrogenase (RALDH) expression (55); and potentially downregulating the production of pro-inflammatory cytokines like IL-6 and IL-12 (56). RA, the active metabolite of vitamin A, not only synergizes with TGF-β to strongly induce Foxp3 expression, promoting naïve T cell differentiation into Tregs, but also guides T cells to express gut-homing receptors (e.g., CCR9 and α4β7 integrin), directing their migration to the intestine (57). It is also worth noting that SCFAs can promote monocyte differentiation into M2-type macrophages with anti-inflammatory and tissue-repair functions (58).

When these metabolite-educated APCs migrate to mesenteric lymph nodes or act directly within the lamina propria, they process and present captured microbial or dietary antigens to naïve CD4+ T cells. In the microenvironment created by the combination of IL-10, TGF-β, and RA provided by APCs, naïve T cells are polarized into Treg cells, thereby maintaining immune tolerance to harmless intestinal antigens (59). Conversely, in the absence of beneficial metabolic signals (e.g., reduced SCFAs and AhR ligands in IBD) or in the presence of strong tissue damage/PAMP signals, APCs may be polarized into a pro-inflammatory phenotype (classically activated M1 macrophages or inflammatory DCs), secreting large amounts of IL-6, IL-1β, and IL-23 (60). The combination of IL-6 and TGF-β drives naïve T cell differentiation toward Th17 (61), while IL-23 is crucial for the expansion, stabilization, and pathogenic conversion of Th17 cells (62). Therefore, by acting as “molecular switches” for APC function, microbial metabolites fundamentally determine whether a local “tolerogenic niche” or an “inflammatory niche” is formed. This represents an early and decisive step in regulating the Treg/Th17 balance. This process underscores the upstream role of microbial metabolic signals in shaping the entire immune response environment, not merely their direct effects on effector T cells.

However, the “educative” effects of different metabolites on APCs exhibit concentration dependence and temporal specificity. For instance, low concentrations of SCFAs may primarily maintain DCs in a tolerogenic state (63), whereas high concentrations or combined signals with AhR ligands may drive a more robust regulatory response. It is also important to note that APC responses to the same metabolic signals may be reprogrammed during different phases of inflammation (e.g., initial vs. chronic stages). In a chronic inflammatory background, APCs may develop resistance to tolerogenic signals like SCFAs (64), a mechanism potentially related to the inhibition of downstream signaling pathways by inflammatory cytokines (e.g., IFN-γ, TNF-α) (65–67). This suggests that future therapeutic interventions need to consider the specific “metabolic-immune context” of the disease stage, rather than simply supplementing metabolites.

### 3.4 Modulation of T cell lineage plasticity and intermediate states

The traditional dichotomy of Treg and Th17 cells as stable, terminally differentiated lineages is an oversimplification. Emerging evidence highlights substantial plasticity between these subsets, characterized by the existence of intermediate or transitional states that co-express or switch lineage-defining factors. These include IL-17^+^Foxp3^+^ cells, regulatory T cells that have lost Foxp3 and acquired pro-inflammatory features (“ex-Tregs”), Th17 cells that gain regulatory functions (“Tr17” or “non-pathogenic Th17”), and cells in transition between states. This plasticity is not merely a pathological artifact but a dynamic layer of immune regulation, and microbial metabolites are poised to be key modulators of this process.

The molecular mechanisms underlying metabolite-mediated regulation of plasticity align with and extend the principles discussed in Sections 3.1 and 3.2. Primarily, metabolites act as environmental cues that calibrate the epigenetic landscape and metabolic fitness of T cells, thereby setting the threshold for lineage commitment and stability. For instance, SCFAs like butyrate, through HDAC inhibition, can promote and stabilize Foxp3 expression by enhancing acetylation at the Foxp3 locus and its enhancer regions. This epigenetic “locking-in” effect may be crucial in preventing Treg cells from losing Foxp3 and converting into ex-Tregs under inflammatory pressure. Conversely, in an inflammatory microenvironment devoid of sufficient SCFAs, the Foxp3 locus may remain epigenetically labile, predisposing Tregs to instability and potential conversion toward a Th17-like or IFN-γ–producing phenotype.

Similarly, AhR ligands play a contextual role in defining Th17 cell fate. As mentioned, AhR activation can skew Th17 differentiation toward a non-pathogenic or regulatory phenotype (producing IL-10 alongside IL-17A) and away from a pathogenic GM-CSF/IFN-γ–producing state. This suggests that AhR signaling does not simply promote Th17 differentiation but actively shapes the quality and plasticity of the resulting Th17 population. In the absence of microbial AhR ligands (as seen in IBD), Th17 cells may default to or more readily acquire a highly pathogenic, unstable phenotype that is more prone to further transdifferentiation.

From a metabolic perspective, the distinct metabolic programs of Tregs (FAO) and Th17 cells (glycolysis) are not just outcomes of differentiation but also active determinants of their plasticity. Metabolites that reinforce a specific metabolic state can stabilize the corresponding lineage. Butyrate, by inhibiting mTORC1 (a driver of glycolysis and Th17 metabolism) and potentially promoting oxidative metabolism, may not only suppress *de novo* Th17 differentiation but also constrain the metabolic reprogramming required for an existing Treg to convert into a Th17-like cell, or for a Th17 cell to stabilize its pathogenic program.

In the context of IBD, the dysregulation of the microbial metabolome creates a microenvironment that actively promotes deleterious plasticity. The depletion of stabilizing signals (SCFAs, AhR ligands) alongside the accumulation of inflammatory cytokines (IL-1β, IL-6, IL-23) and metabolites (succinate) likely lowers the epigenetic and metabolic barriers to lineage conversion. This may facilitate the generation of pathogenic intermediate populations, such as ex-Tregs that exacerbate inflammation or np-Th17 cells that fail to provide barrier protection, thereby contributing to immune dysregulation that is more complex than a simple numerical imbalance. Therefore, a complete understanding of how microbial metabolites regulate intestinal immunity must consider their role in governing the dynamic spectrum and stability of T cell identities, not just their initial differentiation.

## 4 Dysregulation of the microbial metabolic network and immune imbalance in IBD

**Figure 2:**
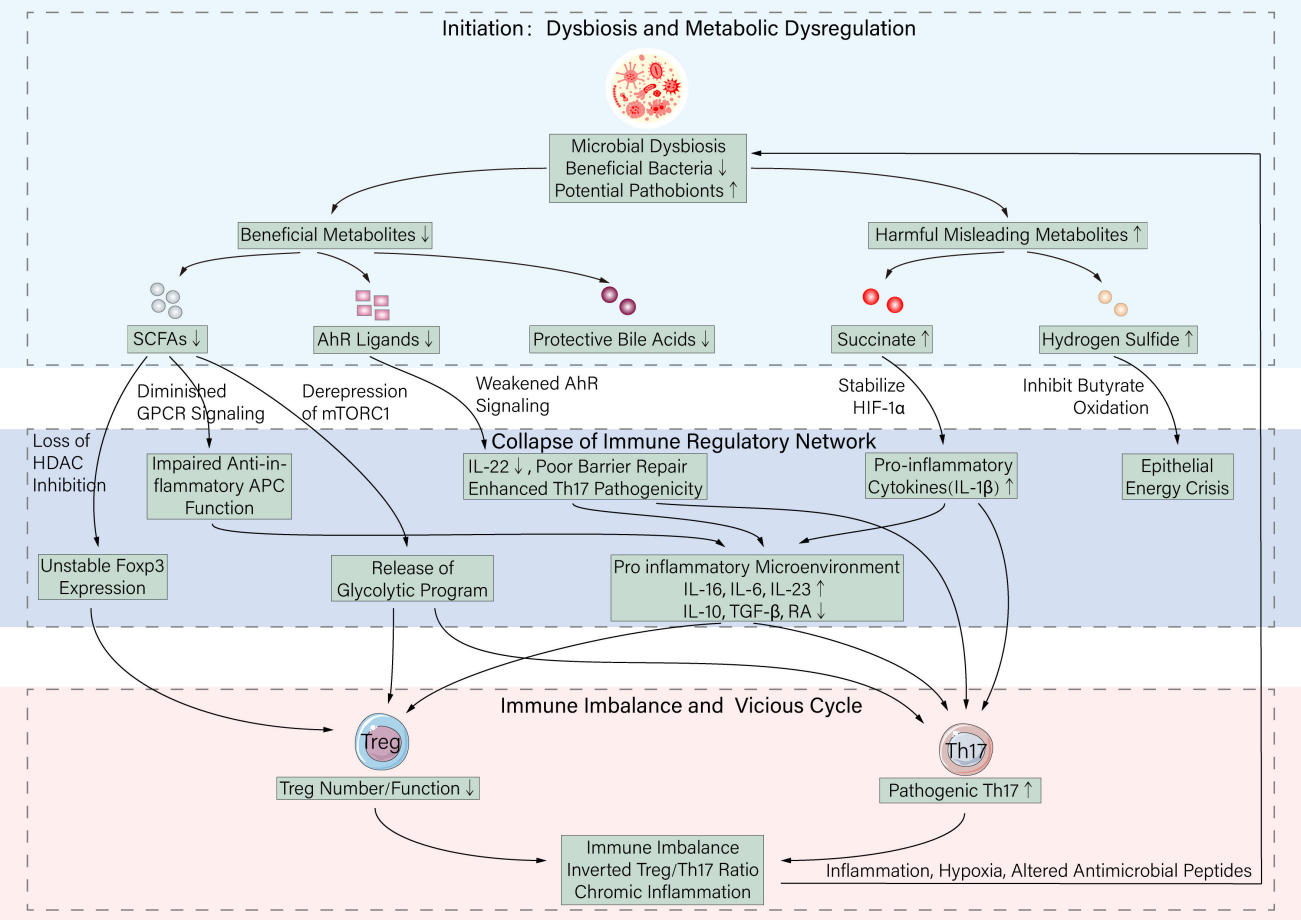
Dysregulation of the microbiota-metabolite network drives Treg/Th17 imbalance in inflammatory bowel disease (IBD). In IBD, intestinal dysbiosis (characterized by reduced beneficial bacteria and expansion of potential pathobionts) leads to a disrupted metabolite profile: beneficial metabolites (SCFAs, AhR ligands, protective bile acids) are depleted, while harmful/misleading metabolites (succinate, H_2_S) accumulate. This metabolic disruption causes collapse of the immunoregulatory network: (1) Reduced SCFAs lead to loss of HDAC inhibition (unstable Foxp3), diminished GPCR signaling (impaired anti-inflammatory APC function), and derepression of mTORC1 (release of Th17-favoring glycolysis); (2) Reduced AhR ligands weaken AhR signaling, impairing IL-22 production and barrier repair while enhancing Th17 pathogenicity; (3) Accumulated succinate stabilizes HIF-1α, promoting pro-inflammatory cytokines; (4) Increased H_2_S inhibits butyrate oxidation, exacerbating epithelial energy crisis. These changes collectively create a pro-inflammatory microenvironment (elevated IL-1β, IL-6, IL-23; reduced IL-10, TGF-β, RA), driving pathogenic Th17 expansion and impairing Treg generation/function. The resulting Treg/Th17 imbalance perpetuates chronic inflammation, which in turn further alters the gut environment (hypoxia, nitrate production, antimicrobial peptide release), exerting selective pressure that worsens dysbiosis and closes the self-perpetuating vicious cycle.

In IBD patients, due to initial genetic susceptibility, long-term environmental factors (e.g., diet, antibiotics), and the reverse effects of inflammation during active disease, the intricate microbial metabolism-immune regulatory network described above undergoes systematic disruption. This dysregulation is characterized not by isolated changes in single metabolites but by a general attenuation of the spectrum of beneficial metabolites with immunosuppressive and tolerance-inducing functions, alongside the relative or absolute accumulation of certain potentially pro-inflammatory or, in this pathological context, detrimental metabolic signals. Together, they synergistically drive a significant tilt of the immune balance toward Th17, entrapping it in a self-reinforcing vicious cycle of chronic inflammation (**Figure 2**).

### 4.1 General attenuation of beneficial metabolic signals: collapse of tolerogenic pillars

Metabolomic studies report significantly reduced total short-chain fatty acids in the feces and colonic mucosal tissues of IBD patients, with butyrate decline being particularly prominent and consistent (68, 69). This is directly related to the depletion of core butyrate-producing bacteria, especially *Faecalibacterium prausnitzii* and *Roseburia* spp (70, 71). *Faecalibacterium prausnitzii* is not only a major butyrate producer but also secretes microbial anti-inflammatory molecules (MAMs); its reduction carries multiple pathological implications (72). The cascade of negative effects from decreased butyrate levels is profound: at the physiological level, colonocytes face an “energy crisis” due to the lack of their primary energy source, leading to reduced synthesis of tight junction proteins (e.g., Occludin, ZO-1), thinning of the mucus layer, impaired goblet cell function, and compromised physical and chemical barrier integrity. This allows increased translocation of microbiota-associated molecular patterns (MAMPs, e.g., LPS) and live bacteria to the submucosa (73, 74). At the immune level, the consequences are more severe. HDAC inhibition in immune cells is lifted, histone acetylation levels at loci like Foxp3 decrease, chromatin closes, resulting in reduced Treg differentiation efficiency and phenotypic instability (75). Concurrently, anti-inflammatory signaling via receptors like GPR109A weakens, diminishing the ability of intestinal macrophages and DCs to produce IL-10 and RA, thus impairing their immune tolerance-inducing function (76). Furthermore, the weakened inhibition of mTORC1 releases the metabolic brake on Th17 differentiation programs, creating conditions for effector T cell overactivation.

Insufficient generation of AhR ligands is another core defect in IBD metabolic dysregulation (77). AhR agonist activity in the intestinal contents of IBD patients is generally reduced, potentially due to a decrease in indole-producing bacteria (e.g., Lactobacillus) or a shift of tryptophan metabolism toward the host’s kynurenine pathway (often upregulated during inflammation) (78). Weakened AhR signaling delivers a dual blow: on one hand, Th17 cells lose a key “taming” signal, making them more prone to differentiate into highly pathogenic subsets producing large amounts of GM-CSF and IFN-γ in inflammatory environments driven by pro-inflammatory factors like IL-23 (24, 79), while their ability to produce the protective factor IL-22 is also impaired (80); on the other hand, reduced AhR signaling in ILC3 cells leads to decreased IL-22 production, directly weakening epithelial repair capacity and antimicrobial defense, further deteriorating barrier function and forming an “epithelial damage-inflammation exacerbation” vicious cycle (81). Additionally, IDO activity may be upregulated due to inflammation, but its products, kynurenine pathway metabolites, may have neurotoxic or immunomodulatory effects, creating complex interactions with the AhR ligand pathway.

Bile acid metabolism also shows characteristic alterations in IBD. Patients often exhibit abnormal bile acid pool composition, including an increased proportion of primary bile acids, altered secondary bile acid profiles, and increased bile acid decoupling (82). Specific secondary bile acids with clear immunosuppressive functions (e.g., 3-oxoLCA) may be reduced, thereby weakening the physiological inhibition of Th17 responses. Moreover, abnormal accumulation of bile acids (e.g., hydrophobic bile acids) may exert direct cytotoxicity on intestinal epithelial cells, exacerbating barrier damage (83). Increased primary bile acids may also overactivate FXR signaling in intestinal epithelial cells, affecting barrier gene expression and cell proliferation (84).

It is important to note that the attenuation of beneficial signals and the accumulation of harmful signals are not isolated events; they often have causal relationships, constituting a chain reaction of network dysregulation. For example, barrier disruption and oxygen influx caused by butyrate reduction (85) directly create a microenvironment favorable for the proliferation of facultative anaerobes (e.g., Enterobacteriaceae) and succinate production (86, 87). Reduced AhR ligand-induced IL-22 secretion weakens epithelial repair, exacerbating leakiness (88). Therefore, primary beneficial metabolic defects may actively drive the initiation of secondary harmful metabolic programs. Understanding these chain relationships helps identify early key nodes for intervening in the vicious cycle.

### 4.2 Generation and accumulation of potentially harmful metabolic signals

Simultaneously, the inflammatory intestinal environment itself creates new selective pressures, altering microbial survival conditions and metabolic strategies, leading to the accumulation of potentially harmful or misleading metabolic signals. Inflammation causes epithelial cells and infiltrating immune cells to produce large amounts of nitric oxide (NO), whose oxidation product nitrate (NO_3_^−^) serves as an efficient electron acceptor, promoting nitrate respiration in facultative anaerobes (e.g., *Escherichia coli* and other Enterobacteriaceae). This gives them a growth advantage over strict anaerobes, leading to their overgrowth in the IBD gut (75). These expanding bacteria may produce different metabolites. For instance, some Enterobacteriaceae produce succinate during respiration. Succinate can stabilize hypoxia-inducible factor-1α (HIF-1α) in inflammatory environments, and HIF-1α not only promotes glycolysis (beneficial for Th17) but also directly enhances the expression of pro-inflammatory cytokines like IL-1β, forming a pro-inflammatory positive feedback loop (89). Succinate can also act as a signaling molecule, promoting inflammatory responses in immune and stromal cells via its receptor SUCNR1 (GPR91) (90).

Under conditions of increased intestinal permeability, plasma protein leakage into the gut lumen also increases, potentially exacerbating bacterial protein fermentation and producing excessive amounts of ammonia, hydrogen sulfide (H_2_S), phenols, and indoles. High concentrations of H_2_S have been shown to inhibit butyrate oxidation in colonocytes, exacerbating the energy crisis, and may induce goblet cell apoptosis, directly damaging the barrier (91). Some protein fermentation products like branched-chain fatty acids and phenylacetic acid may also have pro-inflammatory or cytotoxic properties (92). Together, these changes constitute an environment that exacerbates inflammation and tissue damage at the metabolic level.

### 4.3 Microenvironment remodeling and formation of the vicious cycle

Collectively, the general attenuation of beneficial metabolic signals (SCFAs, AhR ligands, protective bile acids) and the accumulation of potentially harmful signals (succinate, high H_2_S, certain toxic bile acids) in the IBD gut lead to fundamental remodeling of the immune microenvironment in the intestinal lamina propria. This microenvironment shifts from an “tolerance-inducing type” rich in IL-10, TGF-β, and RA to an “inflammation-driving type” rich in IL-1β, IL-6, IL-23, and TNF-α.

In this imbalanced inflammatory microenvironment, APCs are polarized to a pro-inflammatory phenotype, continuously producing cytokines required for Th17 differentiation; naïve T cells are driven toward pathogenic Th17 and Th1 differentiation; and Treg differentiation is suppressed. Their function may also be impaired due to a lack of maintenance signals (e.g., SCFAs, RA) and may even convert into unstable, potentially pro-inflammatory “ex-Tregs” under certain pro-inflammatory cytokines. More critically, this Th17/Treg imbalance and persistent inflammatory state further exert strong ecological selection pressure by altering intestinal physiology (e.g., causing hypoxia, generating nitrate, releasing antimicrobial peptides and ROS). This “selects” and enriches microbiota capable of adapting to and exploiting this inflammatory environment (e.g., oxygen-tolerant, acid-resistant Enterobacteriaceae), while further suppressing strict anaerobic beneficial commensals (e.g., butyrate producers). This leads to aggravated dysbiosis, which in turn worsens metabolic disturbances, forming a self-perpetuating vicious cycle of “dysbiosis-metabolic abnormality-immune inflammation” that is difficult to break.

This cyclic model provides a powerful pathophysiological framework for explaining the chronicity, relapsing nature, and resistance to conventional anti-inflammatory therapies in IBD. Key nodes in the cycle include: (i) IL-23 → pathogenic Th17 → antimicrobial peptide/ROS release → inhibition of strict anaerobes; (ii) hypoxia/HIF-1α stabilization → nitrate generation → Enterobacteriaceae expansion → succinate accumulation → further HIF-1α stabilization and SUCNR1 activation; (iii) barrier damage → plasma protein leakage → abnormal protein fermentation → H_2_S/ammonia production → direct epithelial toxicity and inhibition of butyrate oxidation. These nodes interconnect, forming positive feedback loops. Theoretically, intervening at any node could disrupt the cycle. However, identifying the dominant nodes in different IBD subtypes (e.g., CD vs. UC) or disease stages is a prerequisite for achieving precision therapy. This cycle also suggests that mere anti-inflammatory or immunosuppressive approaches may be insufficient for a cure, necessitating combination with fundamental strategies aimed at restoring beneficial metabolic signals and reshaping microbiota function.

## 5 Therapeutic strategies targeting the microbiota-metabolism-immune axis

Based on an in-depth understanding of how microbial metabolites regulate intestinal immune balance, novel therapeutic strategies aimed at restoring beneficial metabolic signals and correcting immune imbalance are transitioning from laboratory exploration to clinical evaluation. These strategies span a broad spectrum from basic lifestyle interventions to cutting-edge synthetic biology technologies, with a core objective shifting from traditional anti-microbial or immunosuppressive approaches to more fundamental homeostasis restoration and tolerance recovery.

### 5.1 Dietary intervention and precision nutrition

Dietary intervention is the most fundamental, accessible, and impactful method. Epidemiological studies suggest that long-term consumption of dietary patterns rich in fiber (e.g., Mediterranean diet) is associated with a lower risk of IBD (93). The mechanism is thought to partly lie in providing adequate fermentation substrates for colonic commensals, maintaining normal SCFA production. Clinical studies have also observed that increased fiber intake can reshape gut microbiota stability and show symptomatic improvement in some patients with mild-to-moderate UC (94). More forward-looking “precision nutrition” or “precision prebiotic” strategies are emerging. This involves analyzing an individual’s gut microbiota via metagenomics to assess its gene repertoire encoding carbohydrate-active enzymes (CAZymes), thereby predicting its potential to metabolize specific dietary fibers (95). Based on this, tailored combinations of specific dietary fibers that can be preferentially utilized by the patient’s existing beneficial microbiota (e.g., residual butyrate producers) are designed. For example, supplementing arabinoxylan, preferred by *Faecalibacterium prausnitzii*, in patients where this bacterium persists but its function is suppressed, aiming to more efficiently boost endogenous butyrate production, achieving precision fertilization (96).

However, precision nutrition faces a fundamental “chicken-and-egg” dilemma: the gut microbiota function in severe IBD patients is often severely impaired, potentially lacking the initial bacterial species necessary to metabolize specific fibers. Therefore, sequential combination strategies (e.g., first introducing or restoring key metabolic bacteria via FMT or engineered bacterial therapy, then supplementing with their preferred fibers) may be more effective than single interventions. Moreover, adherence to dietary interventions is crucial for long-term efficacy, requiring a balance between personalized recommendations and feasible dietary patterns.

### 5.2 Next-generation probiotics and engineered live biotherapeutic products

Traditional broad-spectrum probiotics (e.g., certain *Lactobacillus* and *Bifidobacterium* strains) have shown inconsistent and generally modest effects in IBD treatment. This is partly because they are often not dominant or key functional species in the complex gut ecosystem, struggling to stably colonize and exert a dominant role (97). Future directions involve developing next-generation probiotics with clearly defined immunomodulatory metabolic functions. This includes directly supplementing commensal bacteria significantly reduced in IBD with clear functions, e.g., *Faecalibacterium prausnitzii* preparations are under clinical evaluation (98). More innovative and promising are engineered live biotherapeutic products. Using synthetic biology tools, bacteria can be genetically modified to stably, efficiently, and controllably produce specific therapeutic metabolites. For example, engineering safe strains like *Escherichia coli* Nissle 1917 to carry key genes in butyrate or propionate biosynthetic pathways, turning them into butyrate-producing factories (99); or engineering bacteria to continuously express AhR ligands or specific bile acid-metabolizing enzymes. These engineered bacteria can be orally administered as living drug delivery systems, reaching intestinal lesion sites to produce high local concentrations of therapeutic molecules. The engineered bacteria themselves may not require long-term colonization, bypassing the ecological niche competition challenges faced by traditional probiotics.

The design of next-generation engineered bacteria needs to move beyond simple constitutive expression toward environmentally responsive intelligent systems. For example, constructing genetic switches induced by harmless signals like tetracycline or thiosulfate to achieve on-demand production and control of therapeutic metabolites. More advanced designs involve developing genetic circuits that sense inflammatory markers (e.g., NO, TNF-α) and feedback-release anti-inflammatory metabolites (e.g., IL-10, TGF-β mimetics, or SCFAs). Such “sense-and-respond” closed-loop systems better mimic physiological regulation, improving safety and reducing off-target effects. However, their long-term safety, genetic stability, immunogenicity, and potential horizontal gene transfer risks still require rigorous evaluation within regulatory frameworks.

### 5.3 Postbiotic therapy: direct supplementation of metabolites

Direct supplementation of chemically defined, optimally formulated metabolite mixtures, i.e., postbiotic therapy, offers another route to circumvent the complexity and uncertainty of live bacteria application. Its advantages include defined composition, controllable dosage, rapid onset, and independence from the host’s microbiota state. Butyrate salts are among the most widely studied postbiotics. Enemas or oral formulations (e.g., tributyrin) have been used in several small-scale clinical trials for mild-to-moderate UC, showing some efficacy in inducing and maintaining remission with good safety (100). A current research focus is developing “cocktail” formulations containing multiple beneficial metabolites, e.g., combining SCFAs, specific AhR agonists (like indole-3-propionic acid), and protective bile acid isomers, aiming for synergistic or additive effects. To ensure these metabolites resist degradation by stomach acid and upper gastrointestinal enzymes and are precisely released in the colon (the primary disease site), developing novel delivery systems is crucial. These include pH-sensitive or colon enzyme (e.g., azoreductase)-responsive microcapsules, liposomes, or nanoparticles. Such technologies can enhance bioavailability, potentially achieve colon-targeted release, and reduce systemic exposure and potential side effects.

### 5.4 Receptor-targeted drug development

From a receptor pharmacology perspective, developing small-molecule agonists or antagonists targeting metabolite receptors is a classic and efficient drug development path. This aims to pharmacologically mimic beneficial metabolite signals or block harmful pathways. For example, synthetic AhR agonists (e.g., Laquinimod) have undergone clinical trials for autoimmune diseases like multiple sclerosis and neurodegenerative disorders (101, 102), and their potential in IBD is being explored. Similarly, high-selectivity agonists for the SCFA receptor GPR109A are in clinical development (103, 104). On the other hand, inverse agonists targeting RORγt, the key driver of Th17 differentiation, represent another promising drug class (105). However, systemic administration suppressing Th17 may increase infection risk, necessitating careful risk-benefit assessment. Local administration (e.g., enemas) or combination with anti-inflammatory drugs may be preferable strategies. In parallel, modulators targeting the bile acid receptor FXR are under development, aiming to regulate bile acid signaling and metabolic inflammation (106).

### 5.5 Optimization and precision of fecal microbiota transplantation

Fecal microbiota transplantation (FMT) aims to holistically restore the recipient’s microbiota structure and its metabolic functions by transplanting the entire microbial ecosystem from a healthy donor. FMT has achieved near-revolutionary success in recurrent Clostridioides difficile infection, but its efficacy in IBD, especially CD, is inconsistent, and long-term safety requires further observation (107). The heterogeneous efficacy is related to multiple factors: donor microbiota quality (richness in beneficial bacteria and metabolic functional genes), severity of recipient intestinal inflammation and ecological niche disruption, and transplantation dose and route. Future optimization is moving toward precision: including functional screening of donors based on metagenomics and metabolomics (e.g., selecting super-donors whose microbiota has high SCFA and AhR ligand production capacity); processing transplant material, such as removing certain components from fecal supernatant or enriching specific bacterial groups/spores; and stratifying recipients (e.g., based on baseline microbiota features, metabolic phenotype, or immune status) to select patient subgroups most likely to benefit.

Beyond screening based on species or metabolomic features, future precision may rely on ex vivo functional screening. This involves co-culturing candidate donor microbiota with peripheral blood mononuclear cells (PBMCs) or autologous intestinal organoids from the recipient patient to directly assess their ability to induce Treg differentiation, inhibit Th17 responses, or promote epithelial repair, thereby selecting the microbiota preparation with the strongest immunomodulatory function. Another promising direction involves the use of defined, “synthetic microbial communities” composed of multiple known beneficial bacteria (e.g., butyrate producers, AhR ligand producers) for transplantation is another promising direction, which potentially improves safety and reproducibility, though it faces challenges in achieving stable coexistence of complex communities *in vivo*.

## 6 Limitations and challenges of current research

Despite significant progress in research on microbial metabolite regulation of the Treg/Th17 axis, the field still faces a series of scientific challenges and translational bottlenecks.

First, establishing causality is difficult. Human studies are mostly observational, making it hard to distinguish whether metabolite changes are a cause or consequence of IBD. Animal models (e.g., germ-free, gnotobiotic mice) can establish causality but differ from humans in complex genetic backgrounds and disease chronicity. Moreover, metabolic networks are complex; changes in a single metabolite often reflect a facet of network dysregulation, potentially overestimating its independent role.

Second, research methods lack sufficient spatiotemporal resolution. Conventional metagenomics and metabolomics analyses disrupt tissue spatial architecture, unable to reveal concentration gradients and dynamic exchange of metabolites across different intestinal regions (e.g., crypts, lymphoid follicles) and cell types (epithelial cells, specific immune cells). There is a lack of fine-grained depiction of metabolite fluctuations longitudinally with disease activity.

Third, there is substantial individual heterogeneity. Host genetics, baseline microbiota, diet, and disease stage lead to vastly different patient responses to the same intervention. Currently, reliable predictive biomarkers to distinguish responders from non-responders are lacking, hindering the realization of personalized precision medicine.

Fourth, intervention strategies face efficacy and durability challenges. Probiotics often fail to stably colonize; dietary interventions require high adherence; long-term safety of engineered bacteria and FMT needs observation; small-molecule receptor agonists may have off-target effects. Converting short-term efficacy into long-term stable remission is a core challenge for clinical translation.

Finally, there is a disconnect between basic mechanistic research and clinical validation. Many targets or therapies effective in animal models often fail to replicate efficacy in complex human trials, highlighting a vast knowledge gap in translating from simplified models to the real human environment.

## 7 Future perspectives

To bridge the gap between current limitations and clinical translation, future research must adopt a more integrated and solution-oriented approach. The following perspectives are structured as targeted pathways to overcome the specific challenges outlined in Section 6.

### 7.1 Deconvolving causality and spatiotemporal dynamics

A primary hurdle is establishing causative links within a complex, spatially organized system (Limitations 1 & 2). Future work must leverage spatial multi-omics technologies (e.g., spatial metabolomics and transcriptomics) to map the precise co-localization of microbial metabolites and immune cell subsets (e.g., Th17, Treg, and intermediate states) within distinct intestinal niches (crypts, lymphoid follicles, lamina propria). This will replace bulk tissue averages with high-resolution “metabolic-immune maps.” Concurrently, longitudinal multi-omics profiling of patients from pre-disease to active and remission phases, integrated with causal inference algorithms (e.g., Mendelian randomization, dynamic network modeling), is essential to move beyond associations and delineate the temporal sequence of dysbiosis, metabolic shift, and immune dysregulation.

### 7.2 Modeling individual complexity for personalized prediction

The profound individual heterogeneity and the failure of animal models to fully recapitulate human disease (Limitations 3 & 5) call for a shift towards patient-centric in silico and *in vitro* models. Constructing personalized systems biology models (“digital twins”) by integrating an individual’s multi-omics data can simulate their unique metabolic-immune network and predict responses to dietary or probiotic interventions. These computational predictions require validation in physiologically relevant human systems. Patient-derived organoid-microbiota-immune cell co-cultures serve as a powerful platform for this purpose, enabling high-throughput, mechanistic testing of therapeutic candidates within a genetically and immunologically matched human microenvironment.

### 7.3 Engineering next-generation context-aware therapeutics

To address the efficacy, durability, and safety challenges of current interventions (Limitation 4), therapeutic strategies must become more sophisticated and adaptive. This involves:

- Intelligent Biotherapeutics: Designing engineered bacteria with genetic circuits that sense local inflammatory biomarkers (e.g., NO, TNF-α) and respond by producing anti-inflammatory metabolites (e.g., SCFAs, IL-10 mimetics) in a feedback-controlled manner.
- Dynamic Precision Nutrition: Moving beyond static prebiotic recommendations to adaptive nutritional guidance based on continuous or periodic monitoring of microbial functional output (e.g., SCFA levels) and host immune markers.
- Sequential and Combination Regimens: Implementing logical treatment sequences, such as using FMT or defined consortia for rapid microbial functional reset, followed by personalized dietary support to maintain the reconstituted ecosystem.

### 7.4 The path to actionable precision medicine

The convergence of the above approaches paves the way for truly actionable precision medicine. The goal is to transition from descriptive “omics” data to clinically predictive biomarker panels. By applying artificial intelligence and machine learning to integrated multi-omics datasets from large, deeply phenotyped patient cohorts, we can identify robust signatures—whether microbial metabolic functions, host metabolite ratios, or immune plasticity profiles—that reliably predict disease course and response to therapies targeting the microbiota-metabolism-immune axis. This will enable a future where treatment is not based on broad diagnosis but on an individual’s functional immune-metabolic network state.

## 8 Conclusion

Gut microbial metabolites play a central role in maintaining the Treg/Th17 balance by integratively regulating immune cell metabolism, epigenetics, and the microenvironment. The development of IBD is closely related to the disruption of this network, entrapping it in a self-reinforcing vicious cycle. Therapeutic strategies targeting this axis, ranging from dietary interventions to engineered live biotherapeutics, show broad prospects for rebuilding immune tolerance at the root. However, the field still faces bottlenecks such as causal complexity, individual heterogeneity, and translational challenges. The future requires leveraging cutting-edge technologies and thinking—including spatial multi-omics, synthetic biology, systems modeling, and artificial intelligence—to pursue spatiotemporal dynamics and causal depth in mechanistic understanding and to focus on functional remodeling and personalized precision in therapeutic development, thereby bringing new hope to IBD patients.
